# Recovery of a US Endangered Fish

**DOI:** 10.1371/journal.pone.0000168

**Published:** 2007-01-24

**Authors:** Mark B. Bain, Nancy Haley, Douglas L. Peterson, Kristin K. Arend, Kathy E. Mills, Patrick J. Sullivan

**Affiliations:** Department of Natural Resources, Cornell University, Ithaca, New York, United States of America; Dalhousie University, Canada

## Abstract

**Background:**

More fish have been afforded US Endangered Species Act protection than any other vertebrate taxonomic group, and none has been designated as recovered. Shortnose sturgeon (*Acipenser brevirostrum*) occupy large rivers and estuaries along the Atlantic coast of North America, and the species has been protected by the US Endangered Species Act since its enactment.

**Methodology/Principal Findings:**

Data on the shortnose sturgeon in the Hudson River (New York to Albany, NY, USA) were obtained from a 1970s population study, a population and fish distribution study we conducted in the late 1990s, and a fish monitoring program during the 1980s and 1990s. Population estimates indicate a late 1990s abundance of about 60,000 fish, dominated by adults. The Hudson River population has increased by more than 400% since the 1970s, appears healthy, and has attributes typical for a long-lived species. Our population estimates exceed the government and scientific population recovery criteria by more than 500%, we found a positive trend in population abundance, and key habitats have remained intact despite heavy human river use.

**Conclusions/Significance:**

Scientists and legislators have called for changes in the US Endangered Species Act, the Act is being debated in the US Congress, and the Act has been characterized as failing to recover species. Recovery of the Hudson River population of shortnose sturgeon suggests the combination of species and habitat protection with patience can yield successful species recovery, even near one of the world's largest human population centers.

## Introduction

In the last 100 years, three genera, 27 species, and 13 subspecies of fish have been extirpated from North America [1]. The US government currently lists more fish (101 [2]) as threatened and endangered species than any other vertebrate taxonomic group. A total of 149 [3] species and distinct populations are currently under federal government protection provided by the US Endangered Species Act, and many have been listed for decades. However, none of these fish species or populations have been designated as recovered and delisted in the three decades since passage of the US Endangered Species Act. Five fish species have been removed from the endangered species list: four by extinction and one by taxonomic revision [3]. Independent review of imperiled fishes [4] in North America also concluded that species recovery is lacking. However, data and research findings reported here on the endangered shortnose sturgeon (*Acipenser brevirostrum*) in the Hudson River of New York indicates this population meets government and scientific criteria for recovery.

The shortnose sturgeon was formally protected with the passage of the 1966 US Endangered Species Preservation Act and later designated as endangered under the current 1973 US Endangered Species Act [5]. The species was considered to be in peril or absent in coastal rivers throughout its range due to overfishing, pollution, and habitat losses from river damming. It is also on the IUCN (International Union for Conservation of Nature and Natural Resources) Red List of Threatened Species [6] because of reduced population size, decline in range and number of locations, and continued decline. Evidence reported here suggests this charter member of the US Endangered Species Act is the first fish to clearly merit designation as a recovered distinct population. The nature of the species, its habitat, and the evidence for a large and secure population is reported as an example of successful protected species management.

The shortnose sturgeon inhabits rivers along the North American Atlantic coast, from the Saint John River, New Brunswick to the St. John's River, Florida. The shortnose sturgeon is best described as an amphidromous [7] species because its use of marine waters is limited to the estuaries of natal rivers [8]. Captures in coastal marine waters and non-natal rivers have occurred but are rare. A long-lived species, shortnose sturgeon maturity is attained in 8 to 10 years and adults may live for 60 years or more [9]. Shortnose sturgeon occupy the lower Hudson River: 246 kilometers of tidal freshwater river and brackish estuary habitats. From late spring through early fall, shortnose sturgeon are dispersed throughout the deep, channel habitats of the freshwater and brackish reaches of this river-estuary [9]. Diet includes insects and crustaceans with mollusks being a major component (25 to 50% of the diet; [10], [11]). In the late fall, most or all adult shortnose sturgeon congregate at a single wintering site near Sturgeon Point (river kilometer, rkm, 139). These fish migrate upstream to spawn in the spring and later disperse throughout much of the estuary.

Hudson River shortnose sturgeon spawn in the spring (late-April to early Mary) downstream of the Troy Dam [9] where the river turbulent and relatively shallow. Eggs adhere to the river bottom, as do the newly hatched larvae [12], [13]. Hatching size ranges from 7 to 11 mm total length (TL; [12], [13]), with Hudson River larvae ranging in size from 15 to 18 mm TL at 10 to 15 days of age [14]. After hatching, larvae gradually disperse downstream over much of the Hudson River Estuary [15]. Larval shortnose sturgeon captured in the Hudson River were associated with deep waters and strong currents [14] , [15].

Juvenile shortnose sturgeon (2–55 cm TL), use a large portion of the tidal reach of the Hudson River. Yearling juvenile sturgeon grow rapidly (to 30 cm TL in first year) and disperse downriver to about rkm 55 by fall [16]. Juvenile distribution during the summer centers on the mid-river region [17] and shifts downriver (Haverstraw Bay, rkm 55–63 [16], [17]) for the late fall and winter seasons.

## Methods

**Figure 1:**
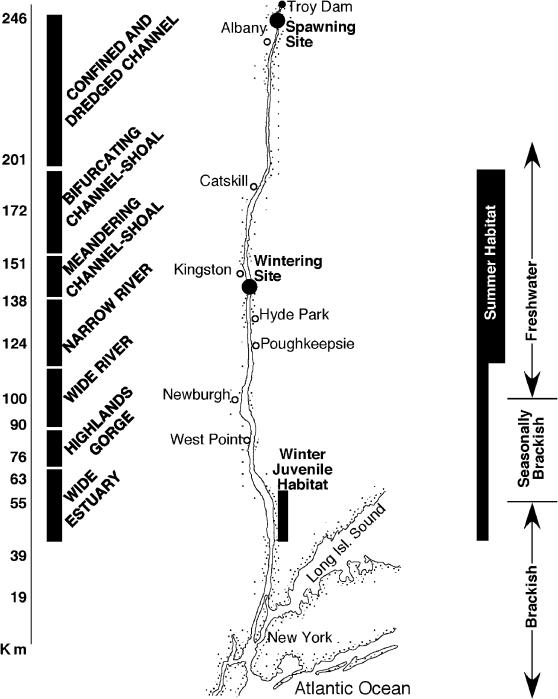
Map of the Hudson River estuary with key habitats used by shortnose sturgeon and the salinity zones in the system. Summer habitat, winter juvenile habitat, and salinity zones match horizontally on the figure with locations in the river. The width of the summer habitat designation corresponds with most and least heavily used sections of the river.

From the Battery in New York (rkm 0) to the Troy Dam above Albany (rkm 246), the Hudson River (Figure 1) spans a river-estuary gradient providing tidal habitats that include freshwater river channels, a brackish fjord, and a rock confined estuary [18]–[20]. Although largely a glacially scoured channel, the Hudson River estuary varies inversely in width relative to depth; maximum width is 4.8 km (rkm 50) while the maximum depth is 66 m (rkm 81). The U.S. Army Corps of Engineers maintains a navigation channel depth of 9 to 11 m although much of the channel in much deeper [20]. Mean ebb and flood current velocities are 0.4 m/s and 0.36 m/s, respectively. The normal tidal amplitude ranges from 0.82 to 1.43 m causing a tidal volume (mean 5,670–8,500 m^3^/s depending on location) from 10 to 100 times river discharge (mean 623 m^3^/s; [20]). Saltwater intrusion extends from rkm 80 to 100 during the summer months (Figure 1) and varies with river discharge. Generally, the limnetic zone (<0.3l ppt) occurs upriver of rkm 80 (Croton Point). An oligohaline zone (0.3–5 ppt) ranges from rkm 40 to 80 with higher salinity (5–18 ppt, mesohaline) further downstream. Sediment characteristics of the Hudson River channel vary along the estuary from sand (dominant above rkm 164) to silty sand (rkm 164 to 148) to clayey silt (below rkm 148 to 64). Larger shell fragments and sandier sediments comprise a larger percentage of channel sediments below rkm 64. Isolated patches of coarser material (sand, gravel) occur near tributary mouths, within the Hudson Highlands, and near Peekskill.

Data on the shortnose sturgeon population in the Hudson River estuary were obtained from a field study we conducted from 1994 to 1997, a shortnose sturgeon population study conducted by William Dovel and others during the 1970s [16], and a standardized fish monitoring program [21], [22] by the Hudson River electric utilities (Central Hudson Gas and Electric Corporation, Consolidated Edison Corporation of New York, New York Power Authority, Niagara Mohawk Power Corporation, and Southern Energy New York). These studies provide a record of the shortnose sturgeon population spanning almost two decades with thorough population estimates made at the beginning and end of the period, and relative abundance data covering many of the intervening years.

Our shortnose sturgeon sampling was completed in two ways: (1) randomly dispersed sampling from June to mid-September (1995 and 1996) throughout the river when the sturgeon were feeding and widely distributed; and (2) targeted sampling of adult sturgeon at their wintering site in December, March, and early April, and their spawning grounds near Albany from mid-April through May (1994 to 1997, Table 1). For both types of sampling we used gill nets (3 m high by 91 m long) with mesh sizes measuring 5-, 10-, and 15-centimeters (stretch mesh). For random sampling, one gill net of each mesh size was anchored and set perpendicular to shore, positioned between mid-channel and the shoreline, parallel to one another and approximately 30 m apart, and deployed in daylight during slack tides (30 to 90 minutes). Targeted gill netting was done in a similar manner but on some occasions a single net was used because catch often exceeded the time available to safely process the fish.

**Table 1:**
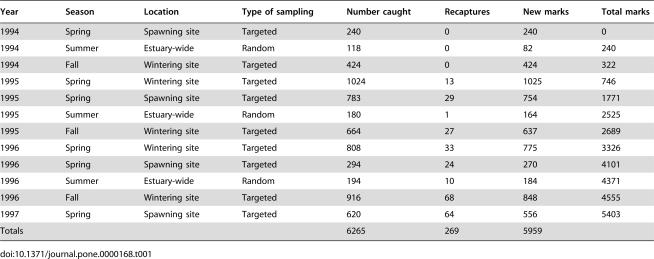
Numbers of shortnose sturgeon marked and recaptured in targeted and random sampling during the study.

Year	Season	Location	Type of sampling	Number caught	Recaptures	New marks	Total marks
1994	Spring	Spawning site	Targeted	240	0	240	0
1994	Summer	Estuary-wide	Random	118	0	82	240
1994	Fall	Wintering site	Targeted	424	0	424	322
1995	Spring	Wintering site	Targeted	1024	13	1025	746
1995	Spring	Spawning site	Targeted	783	29	754	1771
1995	Summer	Estuary-wide	Random	180	1	164	2525
1995	Fall	Wintering site	Targeted	664	27	637	2689
1996	Spring	Wintering site	Targeted	808	33	775	3326
1996	Spring	Spawning site	Targeted	294	24	270	4101
1996	Summer	Estuary-wide	Random	194	10	184	4371
1996	Fall	Wintering site	Targeted	916	68	848	4555
1997	Spring	Spawning site	Targeted	620	64	556	5403
Totals				6265	269	5959	

Fish were removed from gill nets and were either processed immediately on the boat or placed in floating mesh pens along side the boat until being processed. Fish were checked for the presence of PIT (passive integrated transponder) tags, Carlin-Ritchie dangler tags, and Floy tags; PIT tags were applied if one was not present. Fork length (FL) and sometimes total length (TL) were measured to the nearest millimeter and weight measured to the nearest gram. All fish were measured and tagged unless the number of fish caught was so large that processing all of them would take many hours and delay release. At such times, only a subset of the catch was processed, but all were checked for existing tags.

Randomly dispersed sampling occurred between rkm 43 (Tappan Zee Bridge, Nyack, NY) and rkm 246 (Troy Dam) using seven strata based on geomorphological characteristics [18] of the Hudson system. The stratified random sampling design apportioned effort throughout the river. Individual sampling stations (located at river kilometers) were selected using a random numbers table and alternated in orientation to each shore when possible. An equal number of samples were taken in each stratum per month (i.e., June, July, August/September) to ensure equivalent effort throughout the study period.

William Dovel and his associated investigators [16] collected shortnose sturgeon in the Hudson River from 1975 through 1980. Sturgeon were sampled using 6.4 m and 10.7 m otter trawls and drifted, anchored, or staked gill nets of 5.1, 6.4, 7.6, 8.9 cm bar monofilament meshes. Sampling varied among years with trawling occurring between rkm 19 and 246, gill nets between rkm 208 and 246, and some gill net sets below rkm 64. Total or fork lengths were measured to the nearest millimeter and weight was measured to the nearest gram or ounce. Adult and juvenile fish greater than 228 mm TL were marked with Carlin-Ritchie dangler tags attached at the base of the anterior portion of the dorsal fin. Any recaptures were recorded. Sampling in 1979 was conducted from late April through June at the spawning site (rkm 246). For four days each week, two to four drift gill nets were set during slack tide and allowed to drift along the channel bottom for at least 15 minutes [14]. Anchored gill nets were set parallel to shore on both sides of the river in at least six locations each day and allowed to fish overnight. Extensive sampling was conducted between 24 October 1979 and 13 May 1980 at the wintering site near Esopus Meadows (rkm 140; [23]) to capture large numbers of adults.

The standardized fish monitoring program of the Hudson River electric utilities provided annual shortnose sturgeon catch data for years 1985 through 1996. Samples were collected biweekly for 15 weeks from midsummer through fall using a 3.0-m beam trawl. At least three samples were collected in the channel of each of 12 river sampling strata ranging from river rkm 1 through 245 for an annual total of about 1,240 samples. All shortnose sturgeon were recorded with total length in millimeters and weight in grams.

Data analyses were conducted to make comparisons across time and studies, and to provide the best possible population estimates with different data sets. Total length measurements were converted to fork length using the conversion formulae, FL = 0.90(TL) [24], as this relationship corresponds well with TL and FL measurements from double-measured sturgeon in our data sets. Sturgeon less than 500 mm FL were considered juveniles [9]. Fish body condition was calculated using Fulton's Condition Factor K [25], where K(FL) = (weight • 10^5^)/FL^3^.

The shortnose sturgeon population was estimated from mark and recapture data using the Schnabel method that assumes a closed population [26]. This closed population method allowed direct comparison of population estimates from our data and those from the study by Dovel et al. [16], [23]. They also provide precise estimates when assumptions are largely satisfied. Mark and recapture periods were defined by season and location: wintering site in late fall, wintering site in early spring, spawning site in mid to late spring, and summer and early fall dispersed sampling. All marked fish captured in the same sampling period as the period of marking were deleted from the record as recaptures. Multiple recaptures of the same fish were counted as separate recaptures so long as each recapture occurred in separate sampling periods. Comparisons of our estimated population sizes to a population size of 10,000 fish (considered adequate and safe under Endangered Species Act actions for shortnose sturgeon) were made by computing the probability of this observation under our estimated population parameters. A mean and confidence interval for the estimated change in population size between studies was calculated using the distribution of a 1000 randomly selected values from 95% confidence intervals of the population estimates [27].

Closed population estimates assume no significant change in population size occurs during the estimation period due to recruitment, mortality, and movements in or out of the study area. Our study population would not strictly be closed, but shortnose sturgeon are known to be very long-lived fish with low rates of annual mortality and recruitment. Nonetheless, we investigated the potential for bias in our closed population estimates using a series of open population estimates (Jolly-Seber method [26]) and by analyzing the ratio of marked fish in the catch and the known number of marked fish in the estuary through the study period [26]. Finally, we assessed population trend over most of the study period using annual catch rates in the fish monitoring survey of the Hudson River electric utilities.

T-tests were used to test for differences in fish lengths and body condition of sturgeon from our samples and those of Dovel et al. [16], [23]. Paired t-tests were used to determine if there was a significant increase in mean fish length between a series of individual fish marked in the 1970s and recaptured in the 1990s. Differences in fish condition were calculated only from summer catches to avoid potential biases associated with measures of body weights collected immediately prior to or after the spawning season. The dispersed summer distribution of sturgeon was analyzed with a chi-square frequency analysis (samples with and without sturgeon) against a uniform distribution. The presence versus absence data format was used in this analysis so that sites with multiple captures would not bias results.

## Results

**Figure 2:**
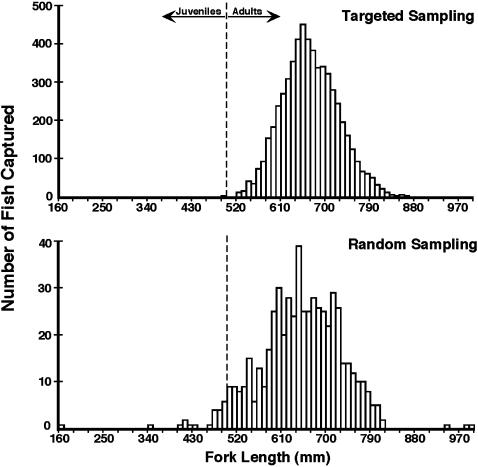
Size distribution of adult shortnose sturgeon captured in targeted sampling in spawning and adult wintering habitats, and the size distribution of shortnose sturgeon captured in random sampling during summer. Shortnose sturgeon greater than 50 cm fork length (FL) were classified as adults. During summer sampling, all life stages of shortnose sturgeon are well distributed in the river system.

We captured 6,265 different shortnose sturgeon and marked 5,959 of these fish. Most (3,836) shortnose sturgeon were captured and marked at the wintering site, high numbers (1,937) were captured and marked at the spawning site, and relatively few (492) sturgeon were handled in the summer random sampling that covered the estuary (Table 1). Recaptures started appearing in the second year of the study (1995) and increased to a total of 269 by the end of our study. Shortnose sturgeon captured during the targeted sampling were adults (Fig. 2), while the summer random sampling captured a broader size range of sturgeon including some juveniles (≤50 cm FL, 4% of total catch).

**Figure 3:**
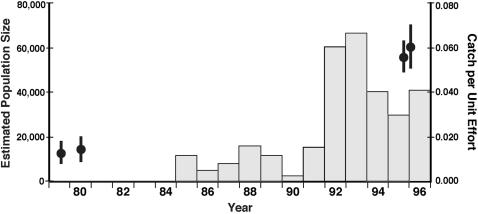
Population estimates and abundance trend for Hudson River shortnose sturgeon in the 1980s and 1990s. The paired symbols of circles (means) and heavy lines (95% confidence intervals) show the results of population estimates in the late 1970s and late 1990s. The catch per unit effort histogram bars are the average catch of shortnose sturgeon per trawl haul in a riverwide fish survey conducted annually by the Hudson River electric utilities.

A closed population estimate (Schnabel method, [26]) based on nine targeted sampling periods yielded 56,708 adult fish with a narrow 95% confidence interval: 50,862–64,072 (Fig. 3). Using the same methods and algorithm, Dovel et al. [16], [23] estimated the number of adult shortnose sturgeon at 12,669 and 13,844 (Fig. 3, 95% confidence intervals of 9,080–17,735 and 10,014–19,224 respectively) in 1979 and 1980. The probability of our sturgeon population was within the range (95% interval) of the Dovel et al. estimates was remote (P<0.001). The population estimates yielded a mean adult sturgeon abundance increase of 407% (95% confidence interval of 290 to 580%) from the late 1970s to the 1990s. Also, the probability that the Hudson River shortnose sturgeon population was 10,000 or fewer fish is highly unlikely (P<0.001) indicating the population was clearly larger than the size considered adequate in Endangered Species Act rulings.

A second closed population estimate was computed using all 12 sampling periods resulting in an estimate of 61,057 shortnose sturgeon with a narrow 95% confidence interval: 52,898–72,191 (Fig. 3). This estimate is larger than the corresponding 9-period estimate, includes juveniles and possibly adults not using the wintering and spawning sites, and is our best estimate of the whole shortnose sturgeon population of the Hudson River. The addition of juvenile and possible non-spawning adult sturgeon in the population was minor (ca. 7% of the overall estimate) indicating that all or nearly all adult shortnose sturgeon are present annually at the overwintering and spawning sites. Also, the summer sampling included some juveniles (4% of total catch) which could account for much of the difference in the 9 and 12 period closed population estimates (Fig. 2).

Analyses addressing the closed population assumption support our population estimates. A regression of the number of marked fish in our targeted sampling catches and the known number of marked fish in the river was linear (R^2^ = 0.96) indicating minimal effect of changing population size during the study. The relation was also linear (R^2^ = 0.84) but less precise when all sampling periods were included. A series of six open population estimates (Jolly-Seber method) varied in results as expected for this method [26] with initial and ending estimates in the series showing high variance. A mid-series set of three estimates had consistent results: population sizes centered on 59,545 with modest variation (coefficient of variation 27 to 30%). Findings using the open population estimates were not different than those using the closed estimates: probabilities of the sturgeon population being 10,000 or fewer fish was remote (P<0.003) and unlikely (P<0.05) to be within the range of the Dovel et al. estimates.

Shortnose sturgeon captured in the 1970s and in our 1990s sampling were very similar in size composition with a slight (mean FL 655 and 665 mm, respectively) but significant (t-test, P≤0.0001) increase in average size. A more equivalent comparison of shortnose sturgeon was made by comparing only those fish captured and measured at the wintering site. In the 1970s, 1,220 captured shortnose sturgeon had a mean fork length of 645 mm while 4,310 sturgeon recorded at the same location in the 1990s had a mean fork length of 663 mm. Again, there was a slight (18 mm) and significant (t-test, P≤0.0001) difference among these large groups of sturgeon. Measures of body condition (Fulton K, [25]) for shortnose sturgeon captured during summers in the 1970s (Mean = 0.845, 95% CI = 0.813–0.877, 13) and 1990s (Mean = 0.835, 95% CI = 0.826–0.845) were similar and are comparable with other populations [24].

Some (37) shortnose sturgeon marked in 1979 and 1980 during the study by Dovel et al. [16] were recovered in our sampling in 1996 and 1997. The fork lengths of these 37 fish after 17 or 18 years in the river indicated very little growth on average (mean increase in FL = 28 mm, P = 0.038). Of these 37 fish, four were juveniles at the time of capture and all of these fish grew (mean increase of 178 mm). There was no increase in length (P = 0.8243) for the 33 sturgeon that were adults when initially measured and marked in the 1970s. Overall, there was very little growth found in fish recovered after 17 to 18 years except for some individuals that were small when initially caught.

From 1985 through 1996, the Hudson River electric utilities conducted an annual trawl survey typically composed of about 1,240 (range 1185–1549) highly standardized samples per year. These data show (Fig. 3) a clear increase in abundance of shortnose sturgeon during the period. Catch ranged from a low of 2 shortnose sturgeon in 1990 to a maximum catch of 82 sturgeon in 1993. The increase in average catch rate was more than four fold higher in the second half of the survey period. The trawl samples captured almost exclusively adult sturgeon with an average total length about 670 mm across years.

Shortnose sturgeon captured during randomly dispersed summer sampling (166 stations, 498 net sets) were distributed non-randomly (X^2^ = 16.87, P<0.01) among seven distinct river strata (Fig. 1). Shortnose sturgeon were most frequently captured (63% of catch, present in 71% of samples) in the middle section of the estuary (Fig. 1, 3 strata from rkm 108 to 189) and were well represented (35% of catch, 51% of samples) in habitats downstream to persistently brackish waters (3 strata from rkm 43 to 107). The primary summer habitat for Hudson River shortnose sturgeon is a deep (regularly 13 to 42 m) tidal freshwater river channel. Downstream the estuary becomes brackish, deeper (regularly 18 to 48 m), and variable in width. The summer distribution of shortnose sturgeon in the Hudson River estuary combined with the wintering and spawning location forms a complete record of major habitats supporting almost all of the population.

## Discussion

Our different population estimates made under varying assumptions indicate a late 1990's shortnose sturgeon population in the Hudson River estuary of about 60,000 fish with adults comprising a very large portion (>90%) of the population. Compared to population estimates in the late 1970s, we conclude the Hudson River population has increased by more than 400%. Independent data from the Hudson River electric utilities annual trawl survey also indicate more than a four fold increase in abundance and again mainly in the adult segment of the population. For the species overall, the Hudson River population is very large and dominant to all others. The number of sturgeon marked during this study exceeds the estimated size of most other populations of shortnose sturgeon [5], and our population estimates are larger than the sum of all other estimated populations. Therefore, it is safe to conclude that Hudson River supports by far the largest population of shortnose sturgeon, and the system may harbor most individuals of the species.

While we assembled multiple lines of evidence supporting a large population increase over two decades, other findings suggest the population of shortnose sturgeon in the estuary is healthy. Shortnose sturgeon captured in the 1970s and in our 1990s sampling were very similar in size composition with a slight increase in average size. Measures of body condition for shortnose sturgeon captured during summers in the 1970s and 1990s were similar and are comparable with other populations [24]. A surprising number of adult sturgeon tagged in the 1970s were recaptured in our 1990s sampling, suggesting that many individual fish have lived for decades in the estuary without growing a measurable amount. These findings depict a population of long-lived fish that has increased in number over decades reaching a high abundance for the species.

Most shortnose sturgeon captured in the Hudson River estuary in research and monitoring programs have been adults ([17], [24], Utilities data set, and this study) regardless of sampling gear and time period. Shortnose sturgeon reach maturity at age-6 or younger with an adult lifespan of several decades [9]. Few unexploited populations of long-lived and large fish have been studied. Some fish populations like this were found to be composed overwhelmingly of slow growing, long-lived adults displaying a normal-shaped size distribution as in Figure 2 [28]. Few young are found in such populations and juveniles slowly add to the adult group, maintaining a very consistent population size structure. Hence, the Hudson River population of shortnose sturgeon displays the characteristics of an unexploited, long-lived fish population.

The availability and security of habitat is an important consideration in US Endangered Species Act decisions. The spawning and wintering habitats of shortnose sturgeon have been well known since the late 1800s when an intense sturgeon fishery operated in the estuary. The juvenile wintering habitat has been described [16], but the spatial extent of summer sturgeon habitat had not been documented. The sections of the Hudson River primarily used by shortnose sturgeon have remained physically intact with shoreline land use established early in the last century. Many historic residential structures and estates are located along the Hudson River, and very limited portions of the waterfront have been used for industrial uses. The spawning site for shortnose sturgeon is removed from the other habitats, because it is centered on turbulent river habitat between the head of tide and the Troy Dam. This section of the Hudson River is surrounded by urban areas and it is immediately upstream of a river section modified to accommodate a port facility. Nevertheless, the spawning site appears to be supporting adequate spawning in its current modified condition.

Section 7(a)(2) of the Endangered Species Act requires Federal agencies to ensure that actions they authorize, fund, or carry out do not jeopardize the continued existence of an endangered species or result in the destruction or adverse modification of critical habitat. The National Oceanic and Atmospheric Administration (NOAA), National Marine Fisheries Service is the responsible federal agency for planning recovery and implementing protection measures for shortnose sturgeon. Since 2000, the NOAA Fisheries Service has reviewed more than 50 proposed actions (e. g., dredging, shoreline stabilization and docks, pollution discharge permits, [29]) potentially affecting shortnose sturgeon in the Hudson River, often specifying protection measures (e.g., construction timing, design changes, local water quality standards). Shortnose sturgeon have also benefited from a cessation of fishing and other harm to individuals by capture, handling, and disturbance. Overall, the approach to recovery of shortnose sturgeon in the Hudson River has been to minimize interference with natural population processes and maintain habitat conditions able to support the species. This protect-and-wait approach to population recovery is in contrast to strategies employed for other species using hatchery-reared fish to actively promote population increases.

The US Endangered Species Act recognizes for listing and delisting populations that are discrete from other populations, and significant in relation to the entire species [distinct populations, 5]. Endangered species recovery plans specify the criteria to remove a species or a distinct population from the list of threatened and endangered species [30] making them key documents defining recovery [31]. The shortnose sturgeon plan [5] names 19 distinct populations and specifies three recovery criteria: adequate size with a favorable trend in abundance; habitat sufficient to support a recovered population; and potential causes of mortality insufficient to reduce the population. A shortnose sturgeon population composed of 10,000 spawning adults has been considered large enough to be at a low risk of extinction by the NOAA [32] and adequate for delisting under the US Endangered Species Act [32], [33]. This population threshold was based on analyses of minimum viable adult population sizes of vertebrates [34] applied to fish [35]. Population viability analysis was found to be an effective and realistic tool for endangered species protection in an analysis of 21 long-term population studies [36]. Other minimum population analyses have identified abundances less than the NOAA criteria for shortnose sturgeon [30], [37]–[42]. Following the criteria used by the NOAA for shortnose sturgeon, our total and spawning population estimates exceed the safe level by a wide margin (≥500%), clearly indicating recovery of this shortnose sturgeon population.

Aside from population size, estuary fish monitoring and the population estimates we report over two decades indicate a positive trend in population abundance. Shortnose sturgeon habitats in the Hudson River have supported the growing and now large population, and both the specific spawning and wintering areas and the widely dispersed growing season habitats have remained intact. No major changes are expected in the tidal portion of the Hudson River that would greatly alter or eliminate deep channel waters or the turbulent spawning reach. Finally, likely future causes of high mortality such as unregulated harvest, bycatch in active fisheries, and pollution stress have been and can be controlled through established fishery management and water quality regulations. By all three criteria specified in the shortnose sturgeon recovery plan, we believe the Hudson River estuary population merits designation as ‘recovered’ and qualifies for delisting from the US Endangered Species Act protection.

The NOAA Fisheries Service periodically reports on the status of shortnose sturgeon throughout their range [5], [43]–[45] using the latest information from field studies. A complex three-river estuary in Maine (Sheepscot, Kennebec, and Androscoggin Rivers) has had increasing numbers (7,222 fish in 1981 to 9,488 in 2000) of shortnose sturgeon recently approaching the safe population size, although there appears to be two distinct spawning populations contributing to the total numbers [46]. Substantial and stable populations occur in the Delaware River (6,408–14,080 in 1981–1984, near 10,000 in 2002, and 8,445 in 2004) and the Saint John River, New Brunswick (18,000 in 1970s). The Connecticut River appears to have a small (<150 fish) stable population isolated above the Holyoke Dam, and an increasing (895 in 1993, 1,800 in 2003[47]) population in the lower river. The Savannah River (South Carolina and Georgia) was stocked with 97,000 shortnose sturgeon between 1984 and 1992 but the most recent population estimate is modest (3,000 in 1999). The large Altamaha River of Georgia supports a modest population (798 in 1990, 468 in 1993, as many as 2,000 in 2004) of shortnose sturgeon. Another 12 mostly small Atlantic coast rivers have some evidence of shortnose sturgeon presence in low numbers (ca.<100) with increasingly frequent captures after decades of no records. Notable is the near lack (18 fish captured since 1996) of shortnose sturgeon in the largest Chesapeake Bay rivers (James, Potomac, and Susquehanna Rivers) although these rivers have dams and obstructions on or close to the tidal zone. What may make the Hudson River unique for shortnose sturgeon is the large area of tidal freshwater habitat used as the summer foraging range: the most commonly occupied 81 km of the tidal freshwater Hudson River. Other rivers with large summer habitat have sizable and near safe level populations (Maine rivers, Delaware River, Saint John River) except in the large southern rivers (Savannah, Altamaha Rivers) where mortality in river gillnet fisheries for shad (*Alosa* spp) is believed a critical impediment [5], [8], [45]. Overall, shortnose sturgeon in the Hudson River and across the species range suggest that slowly increasing populations could reach recovered status where they are managed under full protection in substantial foraging habitat.

Calls to change the US Endangered Species Act have come from scientists and legislators for more than a decade [48]–[50], and changes to this law are being debated in the US Congress [51], [52]. The Act has been characterized as failing to recover species [50], [52], [53], promote effective recovery programs [54]–[56], or properly assess species endangerment [57], [58]. One commonly reported flaw in government species recovery plans is that not enough is being done to increase population size and viability. Foin et al. [58] predict that most (63%) endangered species will not reach recovery criteria through habitat protection alone, and that more active management such as habitat restoration and population augmentation will be needed. Despite the multitude of anthropogenic influences on the Hudson River ecosystem, the shortnose sturgeon population appears to have achieved recovery and may merit removal from the list of threatened and endangered species. Other rivers with shortnose sturgeon appear to be slowly developing larger populations or have impediments that can be addressed with more determined species protection measures. Extension of a protect-and-wait conservation strategy seems viable for recovering shortnose sturgeon populations in the largest un-dammed rivers scattered along the Atlantic Coast.

Another assessment [59] of the Endangered Species Act concludes it is working more often than recognized because of poor reporting on the status and trends of endangered species populations. Few data have been collected following recovery efforts [31], [60], [61] making recovery and species management success difficult to recognize. The population status and trend of shortnose sturgeon in the Hudson River estuary had not been well documented prior to this study. The status of other shortnose sturgeon populations has been widely scattered through time and lacking for about half of the rivers suspected of harboring shortnose sturgeon [5]. More thorough and encompassing assessments of species status and trends could reveal additional recovery successes over time. Such findings provided evidence and optimism that public efforts for endangered species conservation can work. Our analysis of the shortnose sturgeon population in the Hudson River provides the first well documented case that fish species and habitat protection, combined with patience, can result in endangered species recovery; even in a human dominated ecosystem associated with one of the World's largest and most prominent cities.
